# Protein Disulfide Isomerase (PDI1-1) differential expression and modification in Mexican malting barley cultivars

**DOI:** 10.1371/journal.pone.0206470

**Published:** 2018-11-14

**Authors:** Jorge Herrera-Díaz, Mariela K. Jelezova, Felipe Cruz-García, Tzvetanka D. Dinkova

**Affiliations:** 1 Unidad de Servicios de Apoyo a la Investigación y la Industria, Facultad de Química, Universidad Nacional Autónoma de México, Ciudad de México, México; 2 Departamento de Bioquímica, Facultad de Química, Universidad Nacional Autónoma de México, Ciudad de México, México; Murdoch University, AUSTRALIA

## Abstract

Barley malting quality depends on seed characteristics achieved during grain development and germination. One important parameter is protein accumulation in the mature seed, which may vary between cultivars. Here we conducted a protein pattern analysis in the range of p*I* 4–7 of mature grains from five Mexican barley cultivars, commonly used for malt and beer production. Reproducibly distinct protein spots, separated by 2D SDS PAGE, were identified by mass spectrometry and considered as potential markers for cultivars with distinct seed protein accumulation. The expression patterns of glutamate decarboxylase (GAD) and protein disulfide isomerase (PDI1-1) were followed at transcript level during grain development for three independent growth cycles to establish whether differences between cultivars were reproducible. Quantitative determination of PDI1-1 protein levels by ELISA confirmed a reproducibly, distinctive accumulation and post-translational modifications between cultivars, which were independent of plant growth regimes. According to its impact on differential storage protein accumulation, we propose the PDI1-1 protein as potential biomarker for Mexican malting barley cultivars.

## Introduction

Barley (*Hordeum vulgare* L.) is an important cereal used worldwide in food and malt for beer production. The two-row barley (*Hordeum distichon*) is most frequently used in malting, whereas the six-row barley (*Hordeum hexastichon*) is employed in food production. However, in North America the six-row barley is also widely used for malt production [1]. Malting quality depends on seed characteristics achieved during grain development, as well as after germination. Important parameters include the proteins stored in mature seeds and others synthesized and accumulated upon germination [2–3]. Between the last, enzymes required for breakdown of starch, protein and lipid reserves are of utmost relevance. Their catalytic efficiency on available substrates often dictates the optimal timing, temperature and humidity conditions during malting for the upcoming beer production [4–5].

A cereal grain is composed by embryo, scutellum, aleurone layer and endosperm each one playing distinct and important roles during germination [6]. Three main aspects interplay for successful germination: (1) the control on germination and dormancy provided by key proteins synthesized during embryo development; (2) accumulation of storage proteins, lipids, and polysaccharides in the endosperm during seed maturation; (3) a signaling cascade activated by Giberellic Acid (GA) driving synthesis and secretion of α-amylases and other hydrolytic enzyme from the aleurone layer onto the endosperm to partially degrade storage compounds. Although the endosperm cells undergo programmed cell death during seed development, they can exert signals to control embryo growth and the germination process for successful seedling establishment.

A mature barley grain normally contains 9–13% proteins of dry weight. Storage proteins account for most of this content and include prolamins (30–60%), albumin (2.8–12.5%), globulin (2.3–18.1%) and glutelin (7–38%) [7–9]. The barley prolamins, named hordeins, are alcohol-soluble proteins, evolutionarily related to wheat gliadins and glutenins [10]. These proteins usually form a matrix (protein bodies) intercalated with starch granules within the endosperm [11]. Composition and localization of protein bodies could influence their accessibility to hydrolytic enzymes during germination and the malting process [12–13]. Beside hordeins, much of attention has been paid on amylases, hydrolases, proteinases and their inhibitors given their impact on storage reserves degradation during seed germination and direct relationship with the malting process.

Each barley cultivar displays particular malting characteristics depending on its genotype, agronomic management and environmental inputs during growth. In Mexico, spring six-row barley is cultivated for malt and beer production. The *Esmeralda* variety is used primarily under dryland regime, while the *Esperanza* variety is cultivated in irrigated conditions [14]. In an attempt to improve both, yield and malting quality, the Instituto Nacional de Investigaciones Forestales, Agriícolas y Pecuarias (INIFAP) has introduced new spring six-row varieties such as *Adabella* [15], *Alina* [16] and *Armida* [17]. However, the seed protein content for high-yield irrigated cultivars is often above the accepted standards for malt production (13.6–14.6%; S1 Fig). Esmeralda is well adapted to dryland conditions and its grain quality usually meets the required malting parameters, but unfavorable climate conditions could impair yields.

Early studies explored hordein variations in barley varieties [18–19] and some of them tried to correlate the hordein patterns with malting quality [20–21]. Nowadays, availability of genetic markers including those corresponding to the hordein families represents an easier and lesser-time consuming approach to assess the same questions [22–23]. However, distinctive protein expression patterns unveiled through proteomic approaches and activity comparisons between cultivars represent a more faithful portrait of their particular characteristics and have a great impact on the discovery of novel molecular markers [24]. Importantly, molecular markers associated with malting quality, protein composition or stress responses are not available for Mexican barley cultivars, which are the major malt source to produce worldwide distributed and recognized Mexican beer brands. An initial study evaluated the hordein protein patterns in the mature seeds of the varieties cultivated in Mexico, revealing some differences between them [25]. In the same study, more striking differences were found between the malt hordein patterns of cultivars suggesting a distinctive presence or activity of enzymes involved in protein stability/breakdown during germination.

Most storage proteins in barley are synthesized in the rough endoplasmic reticulum (ER) and accumulate in protein bodies derived from the ER and the vacuole [10]. During their synthesis and protein bodies formation they establish essential intra and inter molecular disulfide bonds. Protein Disulfide Isomerase (PDI; EC 5.3.4.1) and related proteins catalyze disulfide bond formation in ER and other cellular compartments [26]. PDI remains present in protein bodies together with chaperones and other accessory proteins to contribute in protein folding, accumulation and even presentation to proteases as evidenced for rice and wheat [27–28]. Hence, its role in the overall storage protein accumulation has been long time predicted [29–30].

Here we explored the global protein patterns in mature grains of five Mexican barley cultivars commonly used for malt and beer production: *Esperan*za (19), *Esmeralda* (04), *Adabella* (11), *Alina* (23) and *Armida* (18). Reproducibly distinct protein spots were observed by two-dimensional electrophoresis separation of proteins from three independent seed batches. Differential spots were identified by mass spectrometry and considered as potential markers to distinguish between cultivars. The expression patterns of selected candidates were followed during grain development for three independent growth cycles to establish whether differences between cultivars are reproducible. According to our results, the major isoform of protein disulfide isomerase (*PDI1-1*) displays reproducibly distinctive accumulation and post-translational modifications between cultivars. Hence, we propose PDI1-1 as candidate biomarker and discuss its potential impact on differential storage protein accumulation in the seeds of Mexican barley cultivars.

## Materials and methods

### Barley cultivars and growth conditions

The Mexican barley cultivars *Esperanza* (19), *Esmeralda* (04), *Adabella* (11), *Alina* (23) and *Armida* (18) were used in this study. All of them are primarily cultivated in Mexico for malt and subsequent beer brewing. For initial protein analyses, field-grown (Spring, 2012) mature seeds were used. Further, independent seed lots were obtained for each cultivar grown under different environments: irrigated, seasonal and greenhouse regimes (2014–2015) to test the conservation of particular protein patterns. For developmental expression analyses, the seeds were cultivated on Metromix 500 substrate in a greenhouse at 22ºC temperature. A nutrient solution (212 ppm nitrogen, 50 ppm phosphorous, 236 ppm potassium, 200 ppm calcium, 48 ppm magnesium, 68 ppm sulfur, 4.25 ppm iron, 2.4 ppm manganese, 0.76 ppm boron, 0.69 ppm copper, 1.35 ppm zinc, 0.012 ppm molybdenum) was added every month to the substrate. Plants were watered every week. Upon spike emergency from the leaf sheath, each spike was labeled 1 DAA (days after flowering). Samples were collected at 1, 5, 10, 15, 20, and 30 DAA, weighted in 0.1 g batches, immediately frozen in liquid nitrogen and stored at –80°C until usage. Three independent biological replicates were analyzed in all experiments.

### Protein extraction

All reagents were purchased from Sigma-Aldrich Química, México unless otherwise stated. For high-yield protein extraction we used a phenol-based method previously standardized for global protein expression analysis during seed development in different plant species [31–33]. Whole barley seeds (500 mg) were first grinded in liquid nitrogen with a mortar and pestle and then homogenized in 10 mL of buffer containing 50% [v/v] phenol, pH 8.8, 0.9 M sucrose, 10 mM ethylene-diamine-tetra-acetic acid (EDTA), 0.4% [v/v] 2-mercaptoethanol, 100 mM Tris-HCl pH 8.8, EDTA-free protease inhibitors (Complete^TM^, Roche Molecular Diagnostics, Pleasanton, California, USA), with an Ultra-turrax homogenizer T-25 (IKA Works Inc., Wilmington, USA). The homogenate was agitated for 30 min at 4°C on a shaker. After a 15 min centrifugation at 10,000 *g*, 4°C, the phenol phase was removed and proteins were precipitated with 5 volumes of ice-cold 0.1 M ammonium acetate in 100% methanol at -80°C for 2 h. The protein pellet was recovered by 10 min centrifugation at 10,000 *g*, washed twice in 10 mL of 0.1 M ammonium acetate in 100% methanol, twice in ice cold 80% acetone and once in 70% ethanol. The pellet was dried for 5 min at room temperature and immediately dissolved in 1 mL isoelectrofocusing (IEF) buffer containing 8 M urea, 2 M thiourea, 4% (w/v) CHAPS, 2% (v/v) Triton X-100, 50 mM dithiothreitol (DTT) and EDTA-free Complete^TM^ protease inhibitors (Roche Molecular Diagnostics). Insoluble matter was removed by centrifugation for 20 min at 18,000 *g*. Protein concentration was determined in triplicate against a standard curve of bovine serum albumin (BSA) using a Bradford based protein assay (BioRad, Hercules, CA).

### Two dimensional gel electrophoresis and analysis

Isolated proteins were separated by two-dimensional (2D) gel electrophoresis as described by [31]. Half gram of total protein brought to 250 μL with IEF was used to hydrate 11 cm IPG strips in a Protean IEF cell unit (BioRad) under the conditions described in [33]. Following IEF, the IPG strips were incubated in SDS equilibration buffer (1.5 M Tris-HCl, 6 M urea, 30% [v/v] glycerol, 5% [w/v] SDS) supplemented with 2% (w/v) DTT for 15 min and then in the same buffer supplemented with 2.5% (w/v) of iodoacetamide. The second dimension was performed on denaturing 12% acrylamide gels in a SE 600 Ruby System (GE Healthcare) until the dye migrated off the gel. The gels were fixed with 50% methanol for 30 min and then, stained with Coomassie Colloidal (20% [v/v] ethanol, 1.6% [v/v] phosphoric acid, 8% [w/v] ammonium sulfate, 0.08% [w/v] Coomassie Brilliant Blue G-250) for at least 16 h.

Two-dimensional gels images were acquired with the ChemiDoc MP system and analyzed with the PDQuest 2-D analysis software version 7.0 (BioRad) to detect, quantify, and match spots. Spots that met the criterion of reproducible changes between the different cultivars in triplicate gels were selected for further analysis. The reference gel was set for cultivar 19. The significance was determined at 0.05 confidence level; p<0.05. According to this, 24 spots were picked from a representative gel of either cultivar for mass spectrometry analysis.

### Mass spectrometry

Mass spectrometry (MS) analysis was performed at the Proteomics and Mass Spectrometry Facility, East State Tennessee University, USA. Protein spots were individually excised from the gel and cut into small (~1 x 1 mm^2^) pieces using a scalpel. The gel pieces were destained, disulfide bonds were reduced, unmodified thiol groups were alkylated, and proteins were digested with trypsin overnight using the In-Gel Tryptic Digestion Kit (Pierce, Rockford, IL) according to the manufacturer’s instructions. The peptides were further extracted from the gel pieces by covering gel pieces with extraction buffer consisting of formic acid/acetonitrile/water (5:50:45, v/v/v) for 10 min. The peptides were completely dried, then rehydrated with 0.1% formic acid and further purified using zip tips (ZipTipU-C18, Millipore Corp., Billerica, MA) according to manufacturer’s instructions. Peptides eluted from zip tips were transferred to vial inserts and completely dried, then rehydrated in a volume of 6 μl of formic acid/acetonitrile/water (0.1:20:79.9, v/v/v). One third of the volume was injected into a C18 picofrit column (New Objective, Inc., Woburn, MA). The column was first equilibrated with 0.1% formic acid in water/acetonitrile (98:2, v/v) and then eluted with a gradient of 2–40% of the solvent containing 0.1% formic acid in acetonitrile. Eluted peptides were analyzed by electrospray ionization LTQ-XL ion trap mass spectrometer (Thermo Scientific, Waltham. MA) and identified with Thermo Proteome Discoverer 1.2 (Thermo Fisher) with a signal to noise threshold of 1.5, precursor mass tolerance of 1400 mmu, and fragment mass tolerance of 0.8 Da.

### RNA extraction

Total RNA was extracted by a modified procedure for endosperm [34]. Briefly, 0.1g of seeds were frozen in liquid nitrogen and crushed by mortar and pestle to a fine powder. The powder was transferred to a two-milliliter Eppendorf tube and 200 μL of extraction buffer (50 mM Tris-HCl pH 8.0; 150 mM LiCl; 5 mM EDTA pH 8.0; 1% SDS) were added. The sample was mixed, extracted twice with 200 μL Phenol-Chloroform and once with Chloroform, centrifuging for 10 min at 10,000 *g*, 4°C each time. The aqueous phase was further extracted with TRIZOL reagent (Life Technologies, USA) in a proportion of 1mL per 100 μL of extract. The RNA was suspended in DEPC-treated water and quantified with Nanodrop. The integrity was determined by 1% agarose gel electrophoresis.

### Reverse transcription and PCR analysis

Previous to the reverse transcription (RT), two micrograms of total RNA were treated with RNase-free DNase (Promega Corp., USA) according to the manufacturer’s instructions. The absence of DNA was verified by a PCR reaction directly performed on RNA (-RT). The RNA was reverse-transcribed with ImProm II reverse transcriptase (Promega Corp., USA) and semi-quantitative PCR was performed using the ribosomal 18S RNA as normalizer. The oligonucleotide primers used in this study were as follows: for *PDI1-1*, forward 5’- *cagcagtggagaggccagtggtcag*, reverse 5’- *ggtacggatggttgtcagggttctt*; for *GAD*, forward 5’- *atggtggtgaccgtggcagcgacg*, reverse 5’- *gcttgccgcactcgggctccatcc*; for rRNA *18S*: 5’- *ggaaacttaccaggtccagacatag*, reverse 5’- *gtggcctaaacggccatagtccctc*. To allow the semi-quantification, the amplification products were followed through cycle progression to determine the final cycle point where linear accumulation was still detected for the product. The PCR products were resolved on 2% agarose gels and the intensity was acquired and analyzed in a KODAK Image Station 4000, Digital Imaging Systems. Densitometry analysis of the amplified products was performed using the program available at the Image Station. Upon background subtraction, band intensity was divided by the area of the band and normalized by dividing the value corresponding to 18S. The earlier developmental stage (1 DAA) was used as control and the rest of stages were expressed as fold of change.

### Immunoblotting

Forty micrograms of total proteins were separated on 10% denaturing polyacrylamide gels (SDS-PAGE) in a Mini-Protean 4 cell (BioRad) and transferred to a PVDF membrane (Millipore). The membrane was blocked with 5% non-fat milk in PBS-T (68 mM NaCl, 1.3 mM KCl, 5 mM Na_2_HPO_4_, 1 mM NaH_2_PO_4_, 1% Tween 20) and incubated overnight at 4°C with a rabbit polyclonal antiserum developed against the barley PDI1-1 carboxi-terminus peptide KAAEPAATEPLKDEL (developed at GenScript USA Inc.) at 1:5000 dilution in the blocking solution. Next, the membrane was rinsed three times with PBS-T for 15 min each, and incubated for one hour with secondary antibodies at 1:10000 dilution in the blocking solution (New England Biolabs, USA). Signal was developed with Luminata HRP Substrate (Millipore, USA) and detected in a ChemiDoc apparatus (BioRad, México). Information about antibody specificities and controls is available in Supporting information (S2 Fig).

### Quantification of PDI1-1 by ELISA

The PDI1-1 synthetic peptide provided by GenScript was used as antigen in calibration curves. The following dilutions were used: 0, 0.1, 0.15, 0.2, 0.25 and 0.3 μg/mL (S3 Fig). Samples were prepared at 1 μg/μL total protein, diluted 1:10 Phosphate Saline Buffer PBS 1X incubated on the plate overnight at 4°C. After three washes with PBS 1X and 1 h blocking with PBS-Blotto in PBS 1X, the plate was incubated with the primary antibody at 1:4000 dilution in blocking solution for 2 h at room temperature. Afterwards, the plate was washed 3 times with PBS 1X and incubated with the secondary antibody conjugated to alkaline phosphatase diluted 1:10000 in blocking solution, for 2 h at room temperature. The signal was detected with p-nitro-phenyl-phosphate at 405 nm. ELISA quantitation was performed in triplicate for three independent biological samples of seeds collected from either field- or greenhouse-grown barley. The PDI1-1 protein levels for each cultivar were referred to cultivar 19.

### PDI1-1 glycosylation analysis

Glycoprotein staining was done with Pro-Q Emerald 300 (Pierce) according to the provider’s recommendations. The CandyCane glycosylated molecular markers were diluted in 1x SDS-PAGE loading buffer to 250 ng of protein per lane. Proteins extracted from different Mexican barley varieties (mature seeds) were separated on SDS-PAGE, transferred to PVDF and detected first for glycosylated proteins and then by immunoblotting against PDI1-1 as described previously in the methods section. Finally the membrane was stained with Sypro Ruby (provided with the Pro-Q Emerald 300 Kit (Pierce). To analyze the glycosylation levels of PDI1-1 the signal detected with Pro-Q Emerald 300 was first normalized to the immunoblot signal and then to the Sypro Ruby signal (for lane). Glycosylation levels for each variety were referred to cultivar 19.

### Statistical analysis

Statistical comparison was done for PDI1-1 quantitative analyses between the Mexican barley cultivars. The data were analyzed by one way ANOVA with Tukey’s multiple comparison and Dunnett’s tests at p < 0.05 at least.

## Results

### Protein 2D-patterns

**Fig 1:**
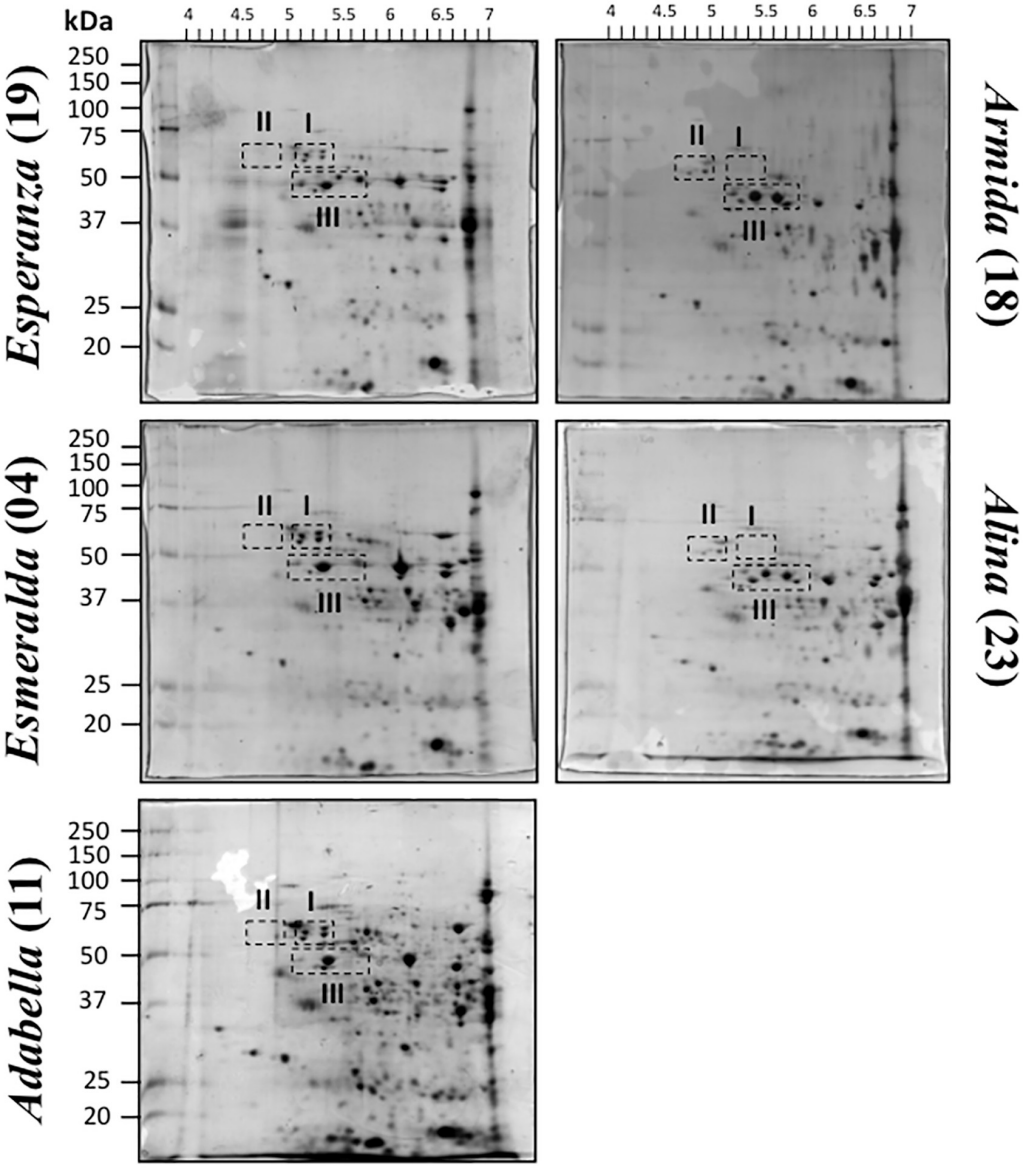
Mature seed protein profiles from five barley Mexican cultivars: Esperanza (19); Esmeralda (04); Adabella (11); Armida (18) and Alina (23). Total proteins were obtained from whole seeds of field-grown cultivars and were separated by 2D gel electrophoresis (range of p*I* 4–7) as described in Methods. Groups of reproducibly different spots between cultivars shown by discontinuous line (I, II, and III) were selected through image analysis with the PDQuest software (BioRad) using as reference cultivar 19.

To analyze the protein patterns of whole barley seeds from five field-grown Mexican cultivars, total protein extracts were fractionated by 2D gel electrophoresis on a narrow range isoelectric focusing (p*I* 4–7) and detected by colloidal Coomassie Brilliant Blue (Fig 1).

**Fig 2:**
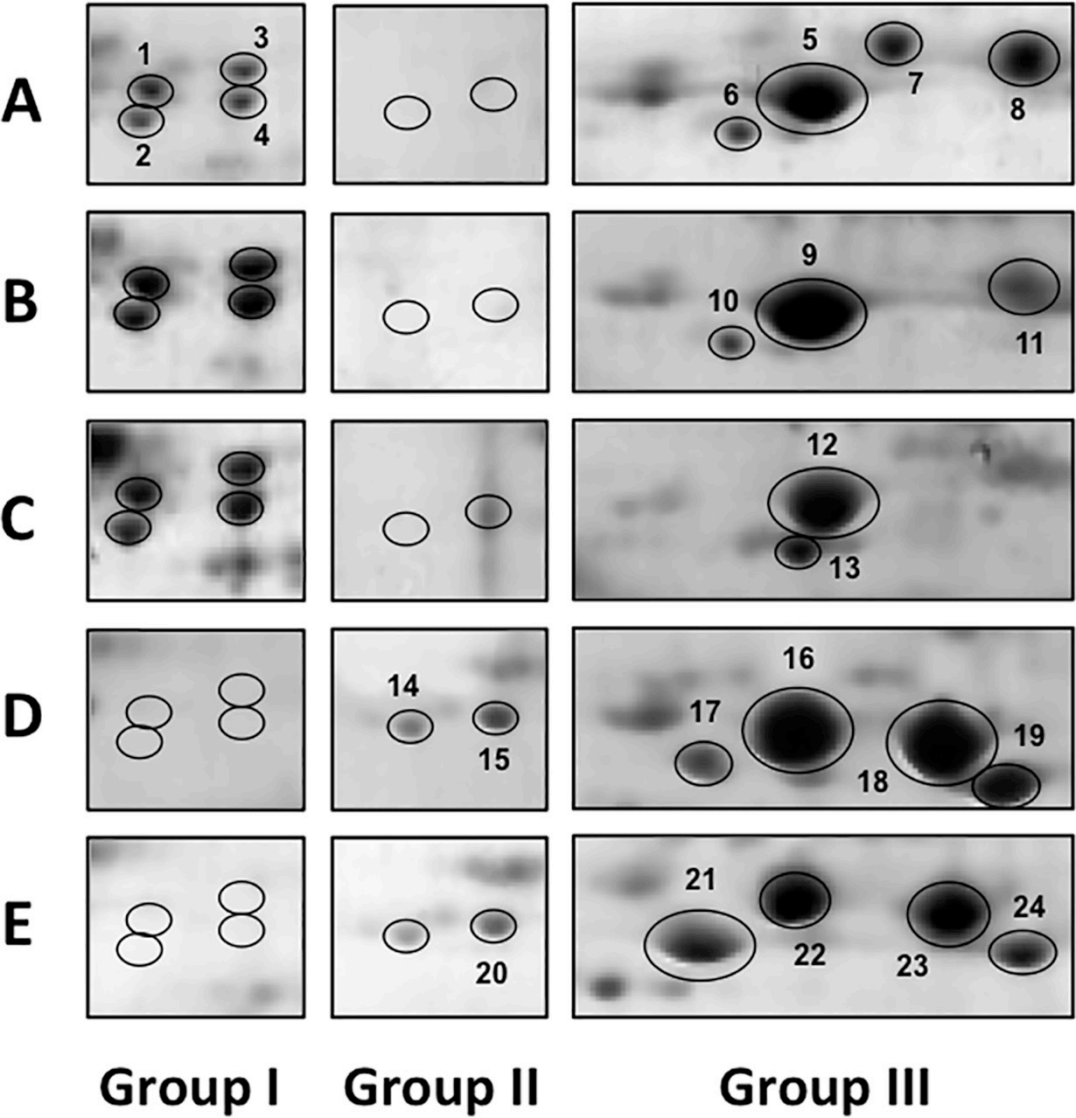
Differential protein spots between five Mexican barley cultivars. Numbered spots for each cultivar: 19 (A); 04 (B); 11 (C); 18 (D); 23 (E) were excised from the gel and identified through LC/MS/MS. Proteins with best score for each spot are reported in Table 1, while the complete list of identified proteins is available in S1 Table.

Although proteins outside p*I* 4–7 were present in seeds form all cultivars, the separation on a wider range (p*I* 3–10) caused poor resolution of the majority of spots detected by Coomassie staining (S4 Fig). Protein extractions and detections were performed by triplicate and all scanned images were analyzed with the PDQuest software as described in the Material and Methods. Protein spots with at least two-fold change and p<0.05 of any cultivar with respect to the reference cultivar 19 were considered for identification. These spots were grouped (I, II, and III) according to their distribution on the gel. A detailed image of the three groups is shown in Fig 2. Group I included four spots that were increased in cultivars 04 and 11 but decreased or absent in 23 and 18. Group II included two spots that were detected only for 23 and 18. Group III was represented by several spots showing reproducibly different levels, either increase or decrease for each cultivar when compared to cultivar 19. Notably, the overall pattern of spot distribution within this group was highly dissimilar between all cultivars.

**Table 1:** Protein identification by MS.

**Spot #**	**Description**	**Short name**	**P**	**Sc**	**MW**	**AC**	**PEP**
**1**	Protein disulfide-isomerase	PDI	1.33E-15	200.31	56427.9	1709617	73
**2**	Protein disulfide-isomerase	PDI	2.66E-14	160.30	56427.9	1709617	27
**3**	Protein disulfide-isomerase	PDI	1.00E-30	90.29	56427.9	1709617	11
**3**	Hsp70	Hsp170	8.27E-13	20.20	66975.0	476003	2
**4**	Protein disulfide-isomerase	PDI	1.38E-07	30.16	56427.9	1709617	4
**4**	Beta-amylase	BAM	9.90E-05	20.14	59609.5	113786	2
**5**	Protein disulfide-isomerase	PDI	2.33E-10	20.23	56427.9	1709617	2
**6**	Disease resistance protein homologue	NBS-LRR	1.20E-02	100.10	152620.4	28555892	39
**7**	Putative transcription repressor HOTR	HOTR1	1.00E+00	24.12	60282.3	3550436	3
**7**	Mildew resistance locus a protein 6	MLA6	1.00E+00	10.15*	107751.0	12957124	9
**7**	Mildew resistance locus a protein 1	MLA1	1.00E+00	10.15*	108539.5	11612210	9
**7**	Mildew resistance locus a protein 6–2	MLA6-2	1.00E+00	10.15*	26749.2	33943715	9
**8**	Elongation factor 1A (Wheat)	EF1A	6.02E-04	20.17	49137.9	399414	2
**8**	RNA polymerase alpha subunit	RPOA	6.40E-03	52.10	38880.3	118430418	24
**9**	UTP-glucose-1-phosphate uridylyltransferase	UDPGP	1.00E-30	30.28	51612.3	6136111	3
**9**	Protein disulfide isomerase	PDI	5.33E-09	50.23	56427.9	1709617	5
**9**	Hypothetical protein						
**9**	ADP-glucose pyrophosphorylase small subunit 1a	AGP-S1a	1.96E-06	20.17	51977.6	1143500	2
**9**	ADP-glucose pyrophosphorylase small subunit 1b	AGP-S1b	1.96E-06	20.17	51962.6	51556842	2
**10**	Mildew resistance locus a protein 6	MLA6	1.00	10.10*	107751	12957124	2
**10**	Mildew resistance locus a protein 1	MLA1	1.00	10.10*	108540	11612210	2
**10**	Mildew resistance locus a protein 6–2	MLA6-2	1.00	10.10*	26749	33943715	2
**11**	Mildew resistance locus a protein 6	MLA6	1.00	10.20*	107751	12957124	7
**11**	Mildew resistance locus a protein 1	MLA1	1.00	10.20*	108540	11612210	7
**11**	Mildew resistance locus a protein 6–2	MLA6-2	1.00	10.20*	26749	33943715	7
**12**	UTP-glucose-1-phosphate uridylyltransferase	UDPGP	1.33E-15	120.29	51612.3	6136111	15
**12**	ADP-glucose pyrophosphorylase small subunit 2	AGP-S2	3.73E-08	20.20	54745.0	27464770	2
**14**	UTP-glucose-1-phosphate uridylyltransferase	UDPGP	7.29E-09	40.17	51612.3	6136111	8
**15**	Protein disulfide-isomerase	PDI	2.04E-09	120.24	56427.9	1709617	20
**15**	Beta-amylase		1.70E-07	40.18	59609.5	113786	4
**16**	UTP-glucose-1-phosphate uridylyltransferase	UDPGP	1.01E-08	60.19	51612.3	6136111	14
**16**	Xylose isomerase	XYLA	2.44E-07	10.18	53549.6	1296807	2
**18**	protein z-type serpin	SrpZ	1.77E-08	40.17	43193.4	1310677	14
**19**	Glutamate decarboxylase	GAD	6.55E-07	40.17	54290.8	31296711	8
**19**	Alanine aminotransferase 2;	ALAAT2	2.52E-05	10.11*	52844.2	1703227	2
**19**	Glucose-1-phosphate adenylyltransferase large subunit 1	AGP-L1	5.01E-05	40.16	57896.0	1707923	8
**19**	ADP-glucose pyrophosphorylase large subunit	AGP-L	8.96E-04	20.12	21231.5	4467847	4
**20**	Protein disulfide-isomerase	PDI	1.11E-16	180.31	56427.9	1709617	32
**21**	ADP-glucose pyrophosphorylase small subunit 2	AGP-S2	9.75E-12	80.22	54745.0	27464770	8
**21**	Glucose-1-phosphate adenylyltransferase small subuinit	AGP-S	9.75E-12	40.22	56013.8	1707940	4
**22**	UTP-glucose-1-phosphate uridylyltransferase	UDPGP	9.19E-13	110.22	51612.3	6136111	26
**22**	Alpha tubulin 5	TUB5	8.14E-11	10.19*	27953.7	146760205	2
**22**	ADP-glucose pyrophosphorylase small subunit 2	AGP-S2	7.81E-10	10.20*	54745.0	27464770	2
**22**	Xylose isomerase	XYLA	7.87E-09	50.19	53579.7	6175480	10
**22**	Glutamate decarboxylase	GAD	1.27E-05	30.19	54290.8	31296711	6
**23**	Glutamate decarboxylase	GAD	3.08E-07	20.13	54290.8	31296711	4
**23**	AF100770_1 receptor-like kinase		2.22E-05	10.14*	45074.1	5669672	2
**23**	Beta-amylase	BAM	4.27E-05	10.12*	59609.5	113786	2
**24**	Glucose-1-phosphate adenylyltransferase large subunit 1	AGP-L1	2.65E-09	30.25	57896.0	1707923	3
**24**	Beta-amylase		3.43E-08	50.19	59609.5	113786	7
**24**	AF414081_1 endosperm-specific beta-amylase 1	BAM-en	3.43E-08	50.19	59534.3	29134855	7

**P:** probability that the protein identification was random (lower number = better identification); **Score:** scoring used in identification algorithm; **MW:** molecular weight reported in accession number; **AC:** NCBI accession number; **PEP:** number of peptides identified for the protein

### Differential spot identification

MS analysis successfully identified proteins in 22 out of the 24 differential spots (Table 1 and S1 Table), albeit some of the identifications displayed low scores (spots #7, #10 and #11). The Spot #13 rendered no match at identification and spot #17 had identifications with less than two peptides. Spots #1–4 rendered the same accession for barley cultivars 19, 04, and 11 representing the protein disulfide isomerase (PDI1-1). Most of the spots had several statistically significant protein identifications according to the probability of a match (P) and the final score (Score; S1 Table). For several spots, the same protein was identified suggesting distinctive p*I* of the same protein, isoforms or association with other proteins, depending on the cultivar. Amongst them were PDI1-1 (spots # 1–5, 9, 15 and 20), β-amylase (spots # 4, 15, 23 and 24) and UTP-glucose-1-phosphate uridylyltransferase (spots # 9, 12, 14, 16 and 22). Other proteins were identified only in specific cultivars, such as Glutamate descarboxylase (GAD) in spots # 19, 22 and 23 from cultivars 18 and 23, z-type Serpin in spot # 18 from cultivar 18, MLA1, MLA6 and MLA6-2 in spots # 7, 10 and 11 from cultivars 04 and 19. It is worth mentioning that MLA1, MLA6 and MLA6-2 identification scores were low (Table 1). However, because of their relevance in barley pathogen resistance, their possibly differential expression in Mexican barley cultivars was considered as important.

### Transcript levels of selected genes during seed development

**Fig 3:**
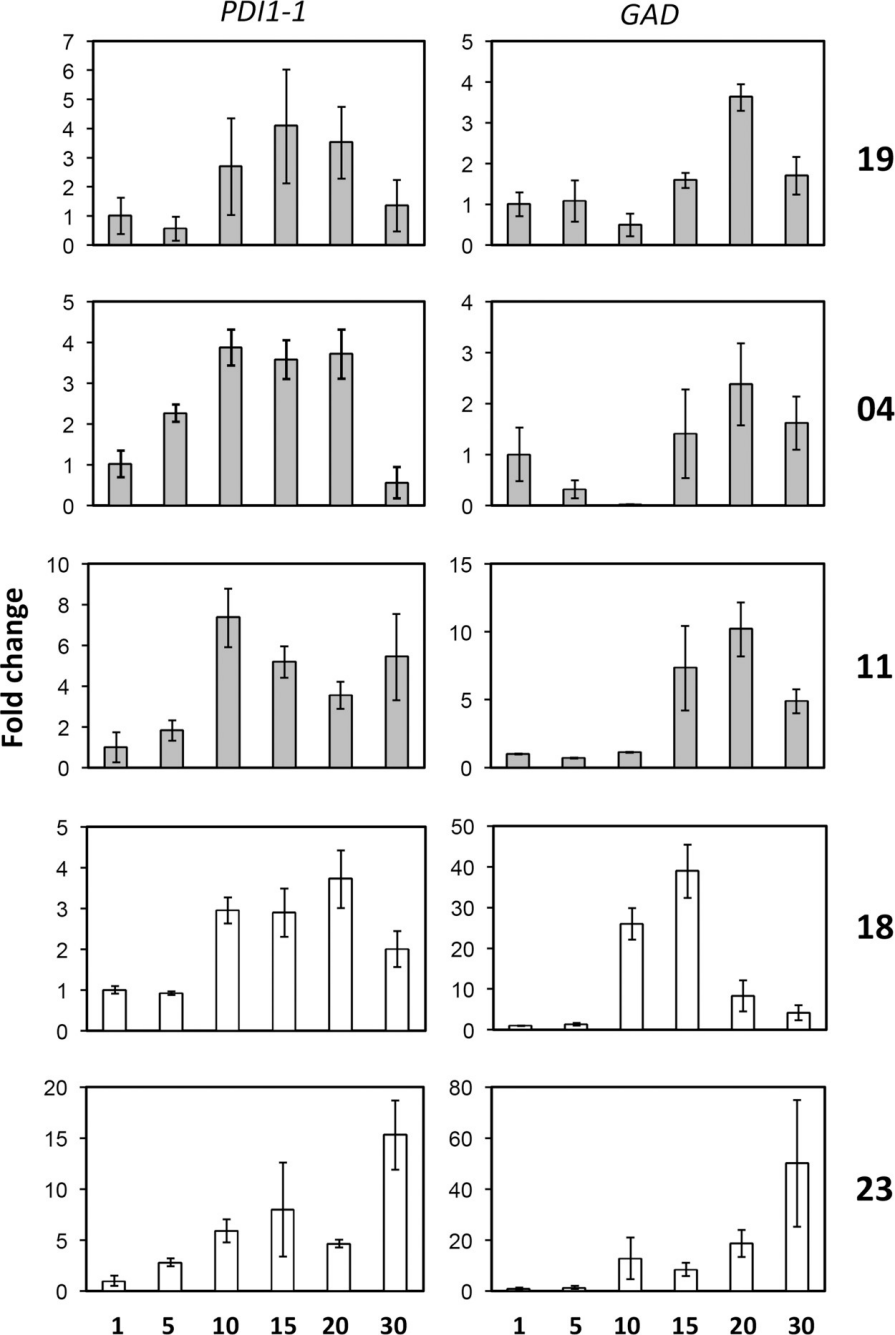
*PDI1-1* and *GAD* mRNA expression levels during grain development. The mRNA levels were analyzed by semi-quantitative RT-PCR using the 18S rRNA as internal reference control. The amplification cycle linearity and densitometry analysis are available in Supporting information, S5 Fig. The results were represented as fold change with respect to the earliest developmental stage (1 DAA) for each cultivar: 19 (A); 04 (B); 11 (C); 18 (D); 23 (E).

The transcript level patterns corresponding to potential biomarkers for Mexican barley cultivars were evaluated. To this end, we performed semi-quantitative RT-PCR analyses for three independent biological replicates of plants grown under greenhouse conditions and tissues collected as described in Materials and Methods. Each biological replicate was tested in triplicates of RNA samples and final point RT–PCR. An example of the semi-quantitative RT-PCR evaluation is shown in S3 Fig. Results from all technical and biological replicates were analyzed by densitometry, normalized to the reference transcript and represented as fold changes with respect to 1 DAA. As shown in Fig 3, the *PDI1-1* and *GAD* transcript profiles differed only between some of the cultivars. For *PDI1-1*, cultivars 19, 04 and 18 displayed transcript increase at 10 DAA but decrease at 30 DAA, while cultivars 11 and 23 increased transcript levels at 10 and 30 DAA. The most striking difference was observed for cultivar 23, where *PDI1-1* reproducibly showed about 15-fold increment at the latest developmental stage. Regarding *GAD*, cultivars 19, 04 and 11 showed similar expression patterns during seed development, different from that observed for either 18 or 23 (Fig 3D and Fig 3E). Again, the increment of *GAD* mRNA was strikingly higher for 23 at 30 DAA.

According to the protein pattern detected for PDI1-1 in the 2D-gels and its transcript level during seed development, a detailed follow up on this protein was performed for the five cultivars through quantitative evaluations.

### Protein disulfide Isomerase (PDI1-1) accumulation in mature seeds of Mexican barley cultivars

**Fig 4:**
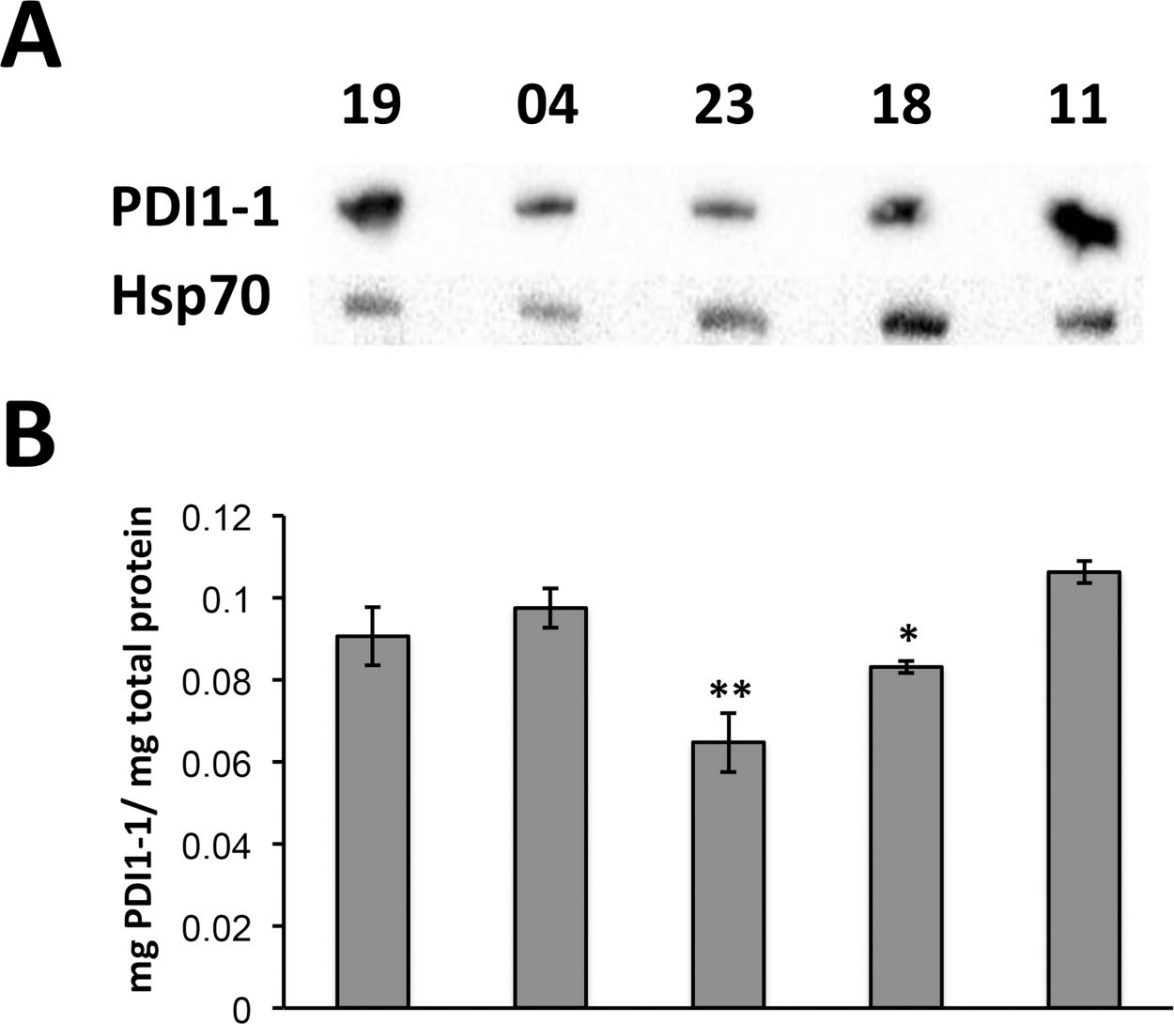
PDI1-1 levels in the mature seeds of five Mexican barley cultivars (19, 04, 23, 18 and 11) grown under field conditions. Proteins were extracted from whole seeds and either separated on denaturing 12% PAGE for western blot analysis (A) or used for ELISA quantitation (B) with antibodies against PDI1-1. For the western blot, Hsp70 was used as reference control. The graph shown in B represents the mean values of PDI1-1 normalized to total protein for three independent protein extractions. Similar quantifications were performed for independent growth conditions (Supporting information, S4 Fig). Significant differences were found for cultivars 23 (**; p<0.01) and 18 (*; p < 0.05).

Specific polyclonal antibodies, developed against the carboxi terminus of barley PDI1-1, were used in Western blot assays. For total proteins from mature seeds, resolved on SDS-PAGE, a single band around 55 kDa was detected for each one of the five cultivars (Fig 4A and S2 Fig). The apparent molecular weight was consistent with the one expected for the full-length protein. The PDI1-1signal was compared to that of Hsp70 as loading control, and reproducibly showed lower intensity for cultivars 18 and 23 in different Western blot comparisons. To perform a quantitative evaluation on the PDI1-1 protein level we standardized ELISA measurements for total protein extracts with antibodies and a PDI1-1 peptide used as antigen (S3 Fig). The PDI1-1 content evaluated by ELISA in triplicate protein extractions and detections from mature seeds showed lower levels for cultivars 18 and 23, reproducing the results observed by Western blot (Fig 4B).

Seed batches used in these evaluations were grown under field conditions. Cultivars 18, 19 and 23 were cultivated under irrigated regime, whereas 04 and 11 were cultivated under seasonal regime. To approach the environmental impact on PDI1-1 accumulation, we tested additional seed batches obtained from either field or greenhouse growth conditions. Regarding the field conditions, either irrigated or seasonal regime was used for all cultivars. Regardless the culture conditions, cultivars 23 and 18 showed significantly lower amount of total PDI1-1 protein content, when compared to 19, 04 and 11 (S6 Fig). Therefore, according to PDI1-1 content, the Mexican barley cultivars fall in two major groups: cultivars 23 and 18 with lower PDI1-1 accumulation in the mature seed and cultivars with higher PDI1-1 accumulation in the mature seed. It was interesting to notice that depending on the growth condition, the overall content of PDI1-1 varied. Seeds obtained from plants grown under irrigated field regime showed the highest accumulation of PDI1-1 (0.2 μg/μg total protein for cultivar 11), whereas those grown under seasonal regime had the lowest accumulation of PDI1-1 (0.07 μg/μg total protein for cultivar 23).

### PDI1-1 patterns of protein accumulation during seed development

The PDI1-1 protein developmental accumulation patterns were tested in all Mexican barley cultivars grown under controlled greenhouse conditions. The grain content was evaluated at 10, 20 and 30 DAA. Selection of these stages aimed to represent grain maturation and dehydration and mirrored the major *PDI1-1* transcript fluctuations (Fig 3) and [35].

**Fig 5:**
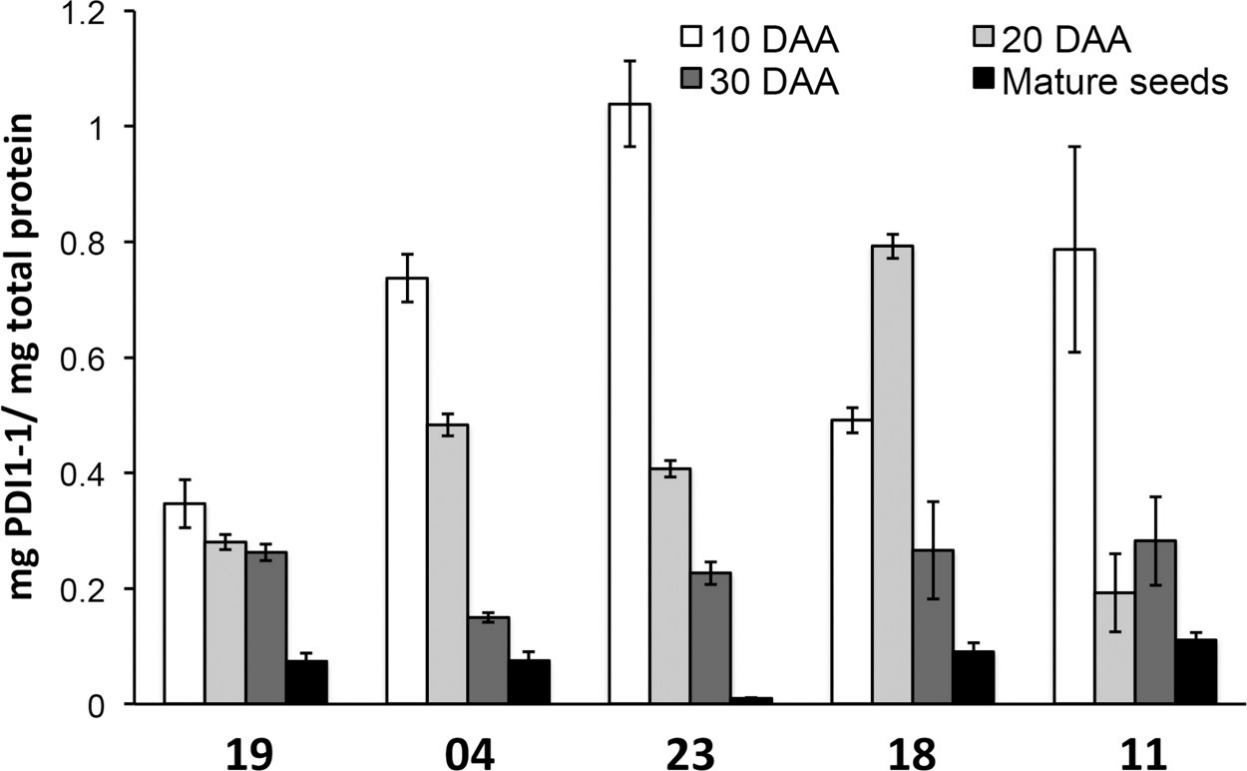
PDI1-1 protein accumulation during grain development. The PDI1-1 protein level was followed at four developmental stages: 10, 20, and 30 DAA, as well as mature seed. The five Mexican barley cultivars (19, 04, 23, 18 an 11) were grown under green house conditions as described in Methods. PDI1-1 quantity was detected by ELISA and normalized to the total protein obtained from whole seeds. The graph represents mean values from two independent growth experiments and three different protein extractions for each experiment.

The results from two independent plant cultures are shown in Fig 5. Four of five cultivars presented the highest PDI1-1 protein level at the earlier developmental stage (10 DAA), which is in agreement with observations for other barley cultivars [35–36]. However, cultivar 18 showed higher PDI1-1 content at 20 DAA. In addition, each cultivar displayed a particular PDI1-1 reduction pattern during grain maturation and dehydration. While for cultivar 19, PDI1-1 was mostly reduced upon dehydration (mature seed vs. 30 DAA), for cultivars 04 and 11 the highest reduction was observed between 20–30 and 10–20 DAA, respectively (Fig 5). Each protein accumulation pattern was reproducible for two independent sowings and growths.

### PDI1-1 glycosylation

**Fig 6:**
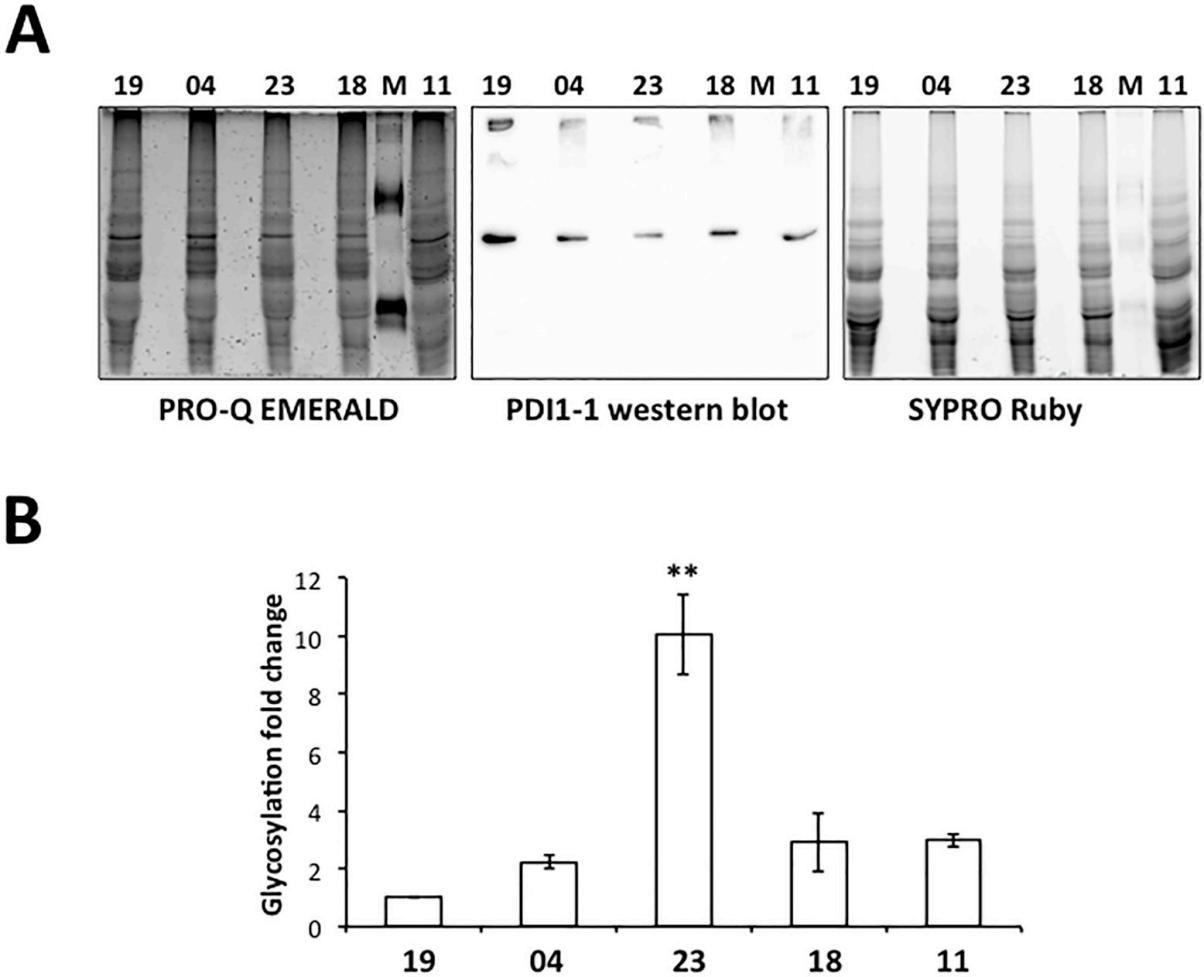
Glycosylation levels of PDI1-1 in the mature seed of five Mexican barley cultivars (19, 04, 23, 18 and 11) grown under field conditions. Proteins were extracted from whole seeds, separated on denaturing 12% PAGE and blotted to a PVDF membrane. The membrane was detected for glycosylated proteins with PRO-Q Emerald staining, total proteins with SYPRO-Ruby staining and western blot for PDI1-1 (A). All the images were superposed to analyze the glycosylation levels of PDI1-1 in each cultivar. The detection was performed for six independent membranes corresponding to three protein extractions. The glycosylation level was estimated according to the densitometry analysis of gPDI1-1/PDI1-1/SYPRO-lane and reported as fold change in cultivars 04, 23, 18 and 11 with respect to the value found for cultivar 19 (B). The analysis from all replicates rendered significantly different glycosylation levels for PDI1-1 from cultivar 23 at p < 0.0001 (***).

The initial proteomic identification of PDI1-1 indicated that the protein localizes at different isoelectric points in the mature seeds of cultivars. For the protein 2-D gels corresponding to cultivars 19, 04 and 11 PDI1-1 was identified in four spots between p*I* 5 y 5.5, while for those from cultivars 23 y 18 the protein was found in spots between p*I* 4.5 y 5, suggesting the possibility of polymorphisms or post-translational modifications varying between cultivars. In an independent protein identification analysis aiming to compare Group I and II spots in cultivars 19 and 23, the mass spectrometry results revealed the presence of glycosylation for this protein (S2 Table). Particularly, PDI1-1 identified from cultivar 23 PDI1-1 was found only with searches within the Uniprot glycosylated protein database. This prompted us to explore the PDI1-1 glycosylation level in the different cultivars. Using the Pro-Q Emerald 300 glycosylation detection approach, PDI1-1 gave positive signal in all cultivars (Fig 6). However, the overall glycosylation level differed between cultivars, when normalized by the Western blot signal and overall protein content (SYPRO Ruby). Particularly, the analysis of independent protein extractions and biological replicates evidenced significantly greater glycosylation for PDI1-1 from in the mature seeds of cultivar 23. Hence, we concluded that mature seeds from cultivar 23 accumulate lower levels of PDI1-1 protein than seeds from other cultivars, but the protein is characterized by its particular glycosylation pattern.

## Discussion

The physical and chemical properties of Mexican barley cultivars commonly used in malt and beer production have been described mostly in agronomical reports [14–17,37]. Such reports describe cultivar features according to the field regime commonly used in their management. However, molecular characterization at protein, RNA, or DNA level is largely missing for any of them. Although some data on starch content and total protein accumulation in the mature seed are available for some of the varieties used in this study [37] and S1 Fig, the evaluation of potential biomarkers responsible for the characteristics of each cultivar or group of cultivars has not been approached.

Protein profiling through 2DE has been largely used in barley seeds employing different protein extraction protocols, range of p*I* in the first dimension and mass spectrometry analyses [2,5]. Instead of describing the whole proteome, we aimed to a deeper understanding of reproducible pattern differences in the proteins accumulated in the mature seeds of five Mexican cultivars. Fine resolution of protein spots in 2DE was preferred against higher spot number detection. Therefore, our analysis included only proteins resolved within the range of p*I* 4–7. According to the protein identification of selected differential spots, enzymes involved in protein processing and carbohydrate metabolism appeared as distinctive between cultivars, at least under the conditions used for this analysis. A protein disulfide isomerase (PDI1-1), glutamate dehydrogenase (GAD) and pathogen-related MLAs, showed contrasting levels between groups of varieties (04 and 19 vs. 18 and 23), whereas other enzymes were identified in all cultivars but at different localization on the 2D gel map.

The analysis of PDI1-1 and GAD transcript levels throughout seed development suggested differential expression patterns for cultivars 18 and 23, although did not reflect the protein differences observed in 2DE. For example, whereas the protein GAD was identified only in differential spots from cultivars 18 and 23, the corresponding transcript was detected for all cultivars and at late seed developmental stages, except for 18, which is consistent with early reports on the GAD presence and activity in barley embryos [38]. This suggests that for seeds from cultivars 04, 11 and 19 the protein might migrate at different position in the gel and even present differential activity. Glutamate decarboxylases catalyze the decarboxylation of Glu to γ-amino-butyric acid (GABA) in a calmodulin-dependent manner [39]. GABA is a four-C non-protein amino acid, generally abundant in plants, that is considered a signaling metabolite in biotic and abiotic stresses, seed maturation and other developmental processes [40]. Importantly, the GABA levels and GAD activity are strongly associated with primary Nitrogen and Carbon metabolisms during seed maturation. A de-regulated GABA accumulation was associated with unbalanced C-N equilibrium in Arabidopsis seeds, and showed considerably increased accumulation of protein at maturity concomitant with lower tri-carboxylic acid cycle intermediates [39]. According to this, it would be worth to measure the GABA levels in seeds from Mexican barley cultivars with contrasting GAD expression patterns and look up for a possible correlation with protein accumulation during maturation.

The reproducibly different localization of spots corresponding to PDI1-1 in cultivars 04, 11 and 19 compared to 18 and 23, as well as their apparently different accumulation levels, prompted us to more deeply characterize this protein as a potential biomarker in Mexican barley cultivars. PDI1-1 is a major protein disulfide isomerase described in barley and wheat during seed development [28,35]. Several genes coding for PDI and PDI-like (PDIL) proteins have been identified in plants but information about their structural and functional features is still limited, reviewed in [41]. These enzymes catalyze the formation of disulfide bonds through their thioredoxin domains. The number and activity of thioredoxin domains varies between members of the family and they have been classified in different phylogenetic groups [26,42].

PDI and PDIL activity becomes very important in cereal grain development, because it is crucial for a correct disulfide bond formation in storage proteins synthesized in the rough endoplasmic reticulum (ER) and accumulated in the endosperm as protein bodies [28–29]. In rice, the lack of PDIL1-1 has been associated with abnormal accumulation of glutelin precursor, defects in the formation of correct prolamin-containing protein bodies derived from ER and the increased accumulation of many types of seed proteins [27,43]. In barley and wheat, PDI1 (PDIL1) represents the most abundant isoform from the reported family members [28,35]. Its levels and localization are thought to exert major influence on the correct protein folding of prolamins and their distribution in storage protein bodies throughout grain development.

The different PDI1-1 accumulation patterns during seed development in the Mexican barley cultivars, as well as their overall seed accumulation levels and post-translational modifications, suggest a correlation with differential prolamin levels and distribution in the mature grain. This would be in agreement with differential protein contents in the seeds from these cultivars, yield and malting characteristics [14]. An important finding in our study was that the differential PDI1-1 protein accumulation in analyzed cultivars was not dependent on the growth regime or geographical region. Although the overall PDI1-1 levels varied for each cultivar according to the growth regime, cultivars 18 and 23 always presented significantly lower amounts of the protein with respect to 04, 11 and 19. This would support its future use as biochemical marker to test in new barley varieties. Furthermore, the differential localization of PDI1-1 on 2DE gels and glycosylation levels evidenced the relevance of post-translational modifications for its stability and/or activity, as previously suggested for rice [27]. According to this, future studies should aim to explore the *in situ* localization of PDI1-1 throughout grain development of contrasting cultivars and its association with protein storage bodies, chaperones and proteases.

In addition to the most closely inspected protein differences described above, our study revealed other proteins with distinct localization/accumulation between the analyzed cultivars. Most of them fall within the carbohydrate metabolism pathways. The relevance of such differences was not further explored here, but might be worth to pursue in the future. It is possible that some of them might result from a different balance between nitrogen and carbohydrate metabolisms due to the GAD levels as previously discussed, or from the effect of PDI1-1 differential levels. However, allelic polymorphisms cannot be ruled out for any of the differentially localized spots identified with the same protein accession.

Further attention might also deserve the expression of MLA proteins identified in spots from only 04 and 19 cultivars. Since the seeds used in the initial proteomic analysis were obtained from different geographic locations, the influence of a potential pathogen presence during the field growth is plausible. In addition, a memory on the expression of these proteins might persist for several generations. All of the cultivars used in this study are considered as moderately resistant to the major barley pathogens affecting the Mexican fields. Therefore, it would be interesting to discern whether they might achieve such resistance through different signaling pathways.

## Supporting information

S1 Fig Dry seed protein content in five Mexican malting barley cultivars. The total protein content was measured by the Kjeldahl method. Four independent seed samples were used for each cultivar. Significant differences were found for cultivars 23 (***; p<0.001) and 18 (**; p < 0.01).
(PDF)

S2 Fig Description and validation of the PDI1-1 antiserum. The carboxi-terminus peptide shown in red was used to generate polyclonal antiserum against PDI1-1 in rabbit (GenScript, USA Inc.). The antiserum was purified against the peptide as recommended by the provider. Different dilutions of the purified antiserum were used in western blot (right upper corner) with total protein extracts from barley seeds (30 μg) and a single band was detected at the expected molecular weight for PDI1-1 (55 kDa). Different amounts (μg) of total protein were tested for the 1:5000 dilution (lower left corner). Immunoprecipitation (IP) with the antiserum showed the same PDI1-1 band, detected in total extracts (T), whereas the pre-immune serum (PI) did not detect the band. High and low molecular weight IgGs were detected in both immunoprecipitate reactions (*). In addition, the band corresponding to PDI1-1 in the IP was identified by mass spectrometry.
(PDF)

S3 Fig ELISA assay calibration for PDI1-1. Different antiserum dilutions and amount of the PDI1-1 immunogenic peptide were used for ELISA calibration reactions in triplicate. The table shows average absorbance for each condition. The dilution 1:4,000 (highlighted) was used for further analyses. The graph below shows a calibration curve of different antigen amounts for the selected dilution, where the range between 0.1 and 0.3 μg showed strong correlation with the absorbance at 405 nm. A calibration curve using antigen amounts within this range was included in each ELISA test performed in the study to calculate the relative PDI1-1 amount in total protein extracts as described in Methods.
(PDF)

S4 Fig Protein profiles in the mature seeds from five Mexican barley cultivars (19, 04, 11, 18 and 23) resolved by 2D-PAGE in the p*I* 3–10 range. (PDF)

S5 Fig Semi-quantitative RT-PCR analysis. Calibration of cycle number for the final point RT-PCR of *PDI1-*1 was performed. The 18S rRNA was used as reference transcript. According to the linearity of the corresponding product increment, 25 cycles were selected for *PDI1-1* and 20 cycles for 18S rRNA. M: molecular marker. On the image below, an example of *PDI1-1* transcript levels are shown. The numbers on the lower image indicate days after flowering. The intensity of each band was corrected dividing by the area and subtracting the negative control (-), which was a reaction without reverse transcriptase. After normalization by the reference control (18S rRNA), the PDI1-1 expression was represented as fold changes with respect to 1 DAF. This analysis was performed in triplicate for each cultivar and for the GAD transcript.
(PDF)

S6 Fig PDI1-1 levels in the mature seeds of five Mexican barley cultivars (19, 04, 23, 18 and 11) under different growth conditions. Proteins were extracted from whole seeds, PDI1-1 quantified by ELISA and normalized to total protein in three independent protein extractions for each growth condition (green house; seasonal regime in the field; irrigated regime in the field). Significant differences were found for cultivars 23 (**; p<0.01) and 18 (*; p < 0.05).
(PDF)

S1 Table Protein identification by mass spectrometry. Twenty-four differential spots were subjected to mass spectrometry identification as described in methods. Proteins highlighted in yellow represent high score and probability, whereas proteins highlighted in green represent high probability but low score in identification. P: probability that the protein identification was random (lower number = better identification); Score: scoring used in identification algorithm; MW: molecular weight reported in accession number; AC: NCBI accession number; PEP: number of peptides identified for the protein.
(XLSX)

S2 Table Protein glycosylation by mass spectrometry. The protein identification was performed according to [15]. Searches were done using Uniprot and Uniprot+Glico databases. The ProteinLynx Global SERVER (PLGS) platform from Waters informatics system indicates confident protein identifications.
(XLSX)
