# Supplementary material for: Protein Disulfide Isomerase (PDI1-1) differential expression and modification in Mexican malting barley cultivars
Source: PLoS One. 2018 Nov 14;13(11):e0206470. doi: 10.1371/journal.pone.0206470 (PMC6235301; doi:10.1371/journal.pone.0206470)
Supplement: S1 Fig — The total protein content was measured by the Kjeldahl method. Four independent seed samples were used for each cultivar. Significant differences were found for cultivars 23 (***; p<0.001) and 18 (**; p < 0.01). (PDF) [file pone.0206470.s001.pdf]

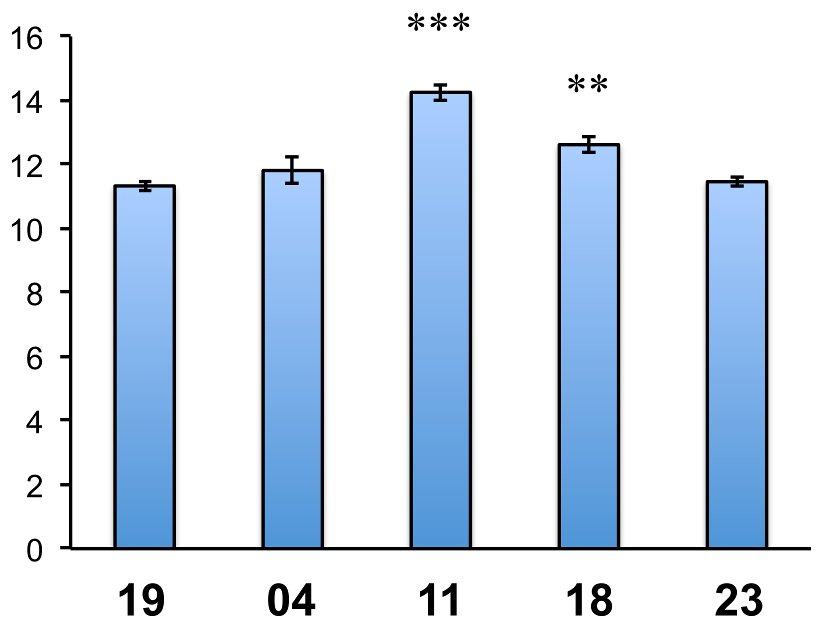

S1 Fig. Dry seed protein content in five Mexican malting barley cultivars. The total protein content was measured by the Kjeldahl method. Four independent seed samples were used for each cultivar. Significant differences were found for cultivars 23 (***; $p<0.001$) and 18 (**; $p < 0.01$).
