# Supplementary material for: Protein Disulfide Isomerase (PDI1-1) differential expression and modification in Mexican malting barley cultivars
Source: PLoS One. 2018 Nov 14;13(11):e0206470. doi: 10.1371/journal.pone.0206470 (PMC6235301; doi:10.1371/journal.pone.0206470)
Supplement: S2 Fig — The carboxi-terminus peptide shown in red was used to generate polyclonal antiserum against PDI1-1 in rabbit (GenScript, USA Inc.). The antiserum was purified against the peptide as recommended by the provider. Different dilutions of the purified antiserum were used in western blot (right upper corner) with total protein extracts from barley seeds (30 μg) and a single band was detected at the expected molecular weight for PDI1-1 (55 kDa). Different amounts (μg) of total protein were tested for the 1:5000 dilution (lower left corner). Immunoprecipitation (IP) with the antiserum showed the same PDI1-1 band, detected in total extracts (T), whereas the pre-immune serum (PI) did not detect the band. High and low molecular weight IgGs were detected in both immunoprecipitate reactions (*). In addition, the band corresponding to PDI1-1 in the IP was identified by mass spectrometry. (PDF) [file pone.0206470.s002.pdf]

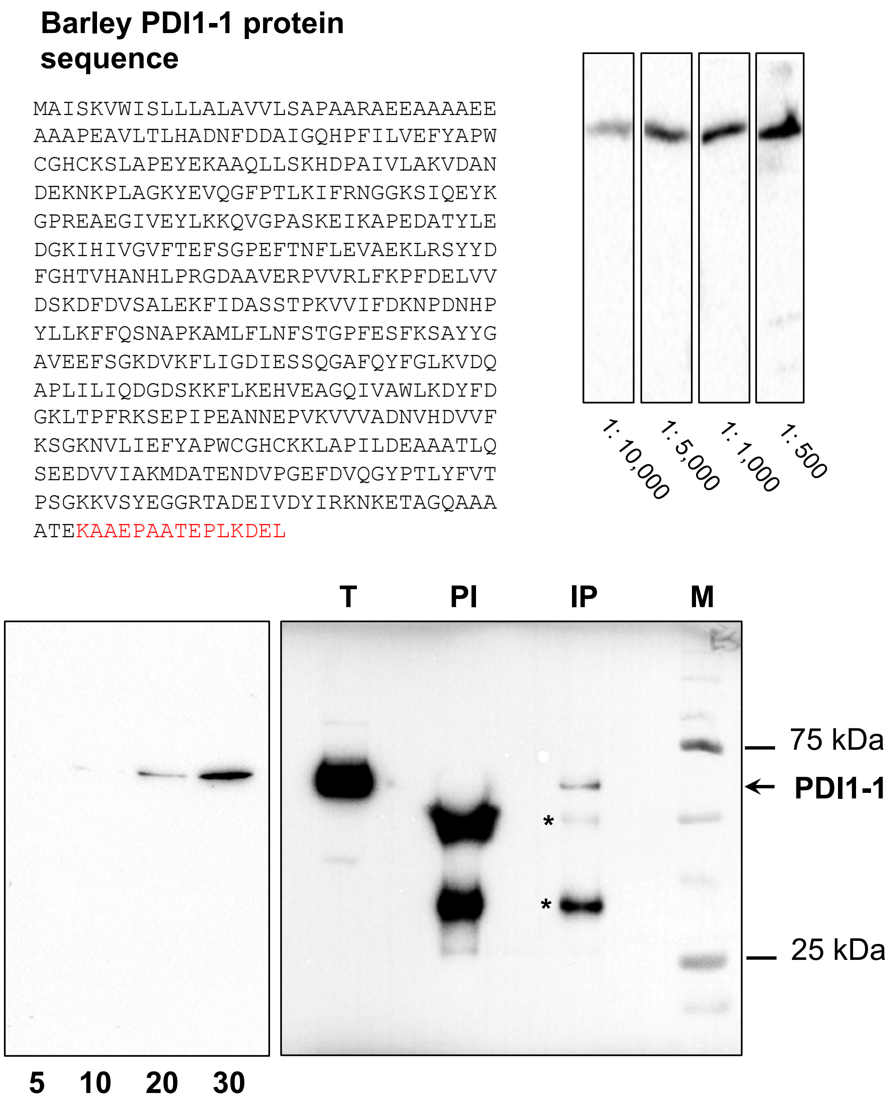

S2 Fig. Description and validation of the PDI1-1 antiserum. The carboxi-terminus peptide shown in red was used to generate polyclonal antiserum against PDI1-1 in rabbit (GenScript, USA Inc.). The antiserum was purified against the peptide as recommended by the provider. Different dilutions of the purified antiserum were used in western blot (right upper corner) with total protein extracts from barley seeds (30 μg) and a single band was detected at the expected molecular weight for PDI1-1 (55 kDa). Different amounts (μg) of total protein were tested for the 1:5000 dilution (lower left corner). Immunoprecipitation (IP) with the antiserum showed the same PDI1-1 band, detected in total extracts (T), whereas the pre-immune serum (PI) did not detect the band. High and low molecular weight IgGs were detected in both immunoprecipitate reactions (*). In addition, the band corresponding to PDI1-1 in the IP was identified by mass spectrometry.
