# Supplementary material for: Protein Disulfide Isomerase (PDI1-1) differential expression and modification in Mexican malting barley cultivars
Source: PLoS One. 2018 Nov 14;13(11):e0206470. doi: 10.1371/journal.pone.0206470 (PMC6235301; doi:10.1371/journal.pone.0206470)
Supplement: S3 Fig — Different antiserum dilutions and amount of the PDI1-1 immunogenic peptide were used for ELISA calibration reactions in triplicate. The table shows average absorbance for each condition. The dilution 1:4,000 (highlighted) was used for further analyses. The graph below shows a calibration curve of different antigen amounts for the selected dilution, where the range between 0.1 and 0.3 μg showed strong correlation with the absorbance at 405 nm. A calibration curve using antigen amounts within this range was included in each ELISA test performed in the study to calculate the relative PDI1-1 amount in total protein extracts as described in Methods. (PDF) [file pone.0206470.s003.pdf]

Peptide (µg)	Antibody dilution						
	1:1,000	1:2,000	1:4,000	1:8,000	1:16,000	1:32,000	1:64,000
**0**	0.057	0.052	0.047	0.057	0.032	0.045	0.051
**0.25**	0.791	0.43	0.266	0.185	0.141	0.126	0.1
**0.5**	1.005	0.603	0.328	0.214	0.147	0.119	0.11
**1**	1.205	0.822	0.543	0.282	0.194	0.134	0.118

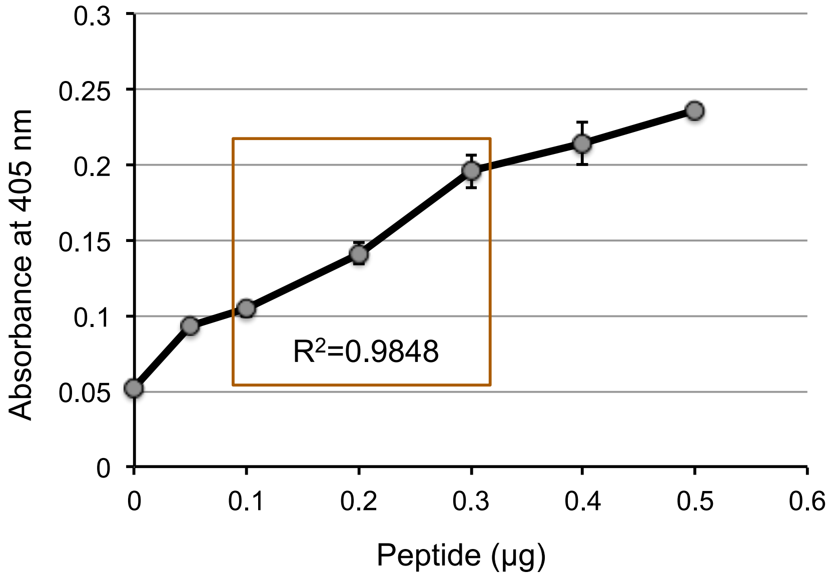

S3 Fig. ELISA assay calibration for PDI1-1. Different antiserum dilutions and amount of the PDI1-1 immunogenic peptide were used for ELISA calibration reactions in triplicate. The table shows average absorbance for each condition. The dilution 1:4,000 (highlighted) was used for further analyses. The graph below shows a calibration curve of different antigen amounts for the selected dilution, where the range between 0.1 and 0.3 µg showed strong correlation with the absorbance at 405 nm. A calibration curve using antigen amounts within this range was included in each ELISA test performed in the study to calculate the relative PDI1-1 amount in total protein extracts as described in Methods.
