# Supplementary material for: Protein Disulfide Isomerase (PDI1-1) differential expression and modification in Mexican malting barley cultivars
Source: PLoS One. 2018 Nov 14;13(11):e0206470. doi: 10.1371/journal.pone.0206470 (PMC6235301; doi:10.1371/journal.pone.0206470)
Supplement: S4 Fig — (PDF) [file pone.0206470.s004.pdf]

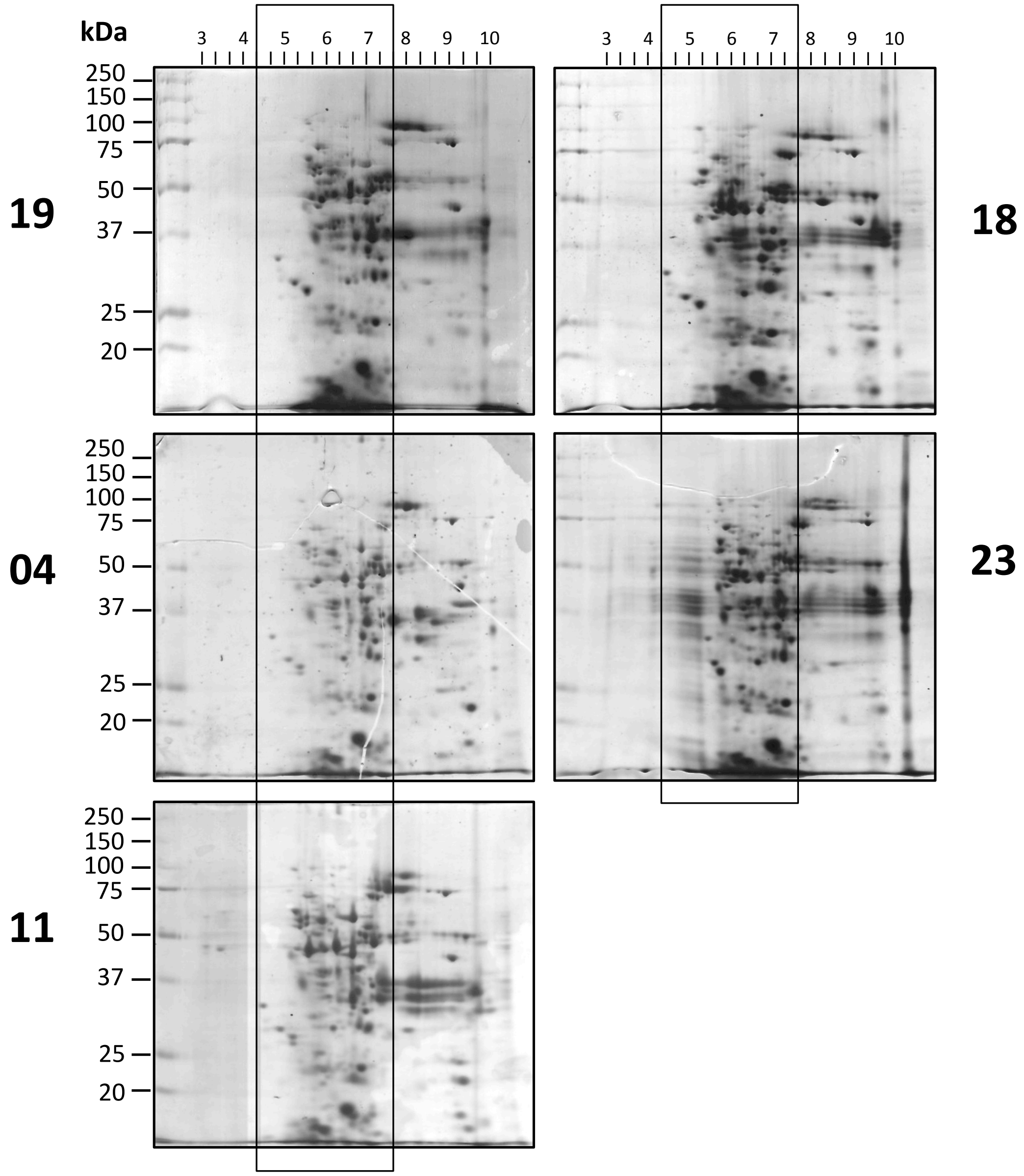


**S4 Fig. Protein profiles in the mature seeds from five Mexican barley cultivars (19, 04, 11, 18 and 23) resolved by 2D-PAGE in the p*I* 3-10 range.**
