# Supplementary material for: Protein Disulfide Isomerase (PDI1-1) differential expression and modification in Mexican malting barley cultivars
Source: PLoS One. 2018 Nov 14;13(11):e0206470. doi: 10.1371/journal.pone.0206470 (PMC6235301; doi:10.1371/journal.pone.0206470)
Supplement: S5 Fig — Calibration of cycle number for the final point RT-PCR of PDI1-1 was performed. The 18S rRNA was used as reference transcript. According to the linearity of the corresponding product increment, 25 cycles were selected for PDI1-1 and 20 cycles for 18S rRNA. M: molecular marker. On the image below, an example of PDI1-1 transcript levels are shown. The numbers on the lower image indicate days after flowering. The intensity of each band was corrected dividing by the area and subtracting the negative control (-), which was a reaction without reverse transcriptase. After normalization by the reference control (18S rRNA), the PDI1-1 expression was represented as fold changes with respect to 1 DAF. This analysis was performed in triplicate for each cultivar and for the GAD transcript. (PDF) [file pone.0206470.s005.pdf]

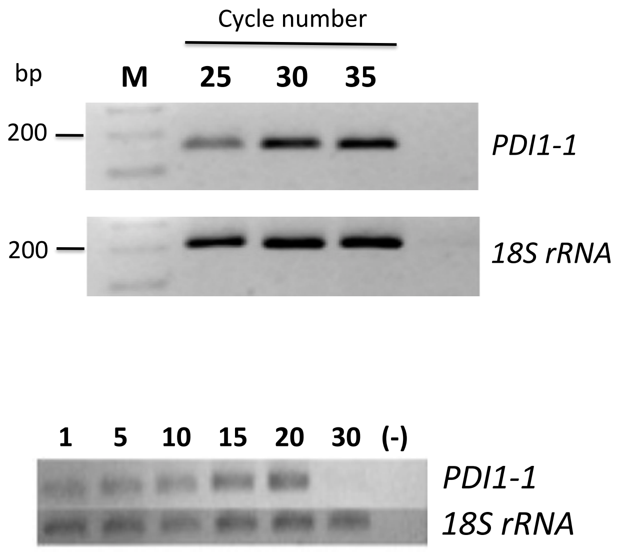

S5 Fig. Semi-quantitative RT-PCR analysis. Calibration of cycle number for the final point RT-PCR of *PDI1*-1 was performed. The 18S rRNA was used as reference transcript. According to the linearity of the corresponding product increment, 25 cycles were selected for *PDI1-1* and 20 cycles for 18S rRNA. M: molecular marker. On the image below, an example of *PDI1-1* transcript levels are shown. The numbers on the lower image indicate days after flowering. The intensity of each band was corrected dividing by the area and subtracting the negative control (-), which was a reaction without reverse transcriptase. After normalization by the reference control (18S rRNA), the PDI1-1 expression was represented as fold changes with respect to 1 DAF. This analysis was performed in triplicate for each cultivar and for the GAD transcript.
