# Supplementary material for: Protein Disulfide Isomerase (PDI1-1) differential expression and modification in Mexican malting barley cultivars
Source: PLoS One. 2018 Nov 14;13(11):e0206470. doi: 10.1371/journal.pone.0206470 (PMC6235301; doi:10.1371/journal.pone.0206470)
Supplement: S6 Fig — Proteins were extracted from whole seeds, PDI1-1 quantified by ELISA and normalized to total protein in three independent protein extractions for each growth condition (green house; seasonal regime in the field; irrigated regime in the field). Significant differences were found for cultivars 23 (**; p<0.01) and 18 (*; p < 0.05). (PDF) [file pone.0206470.s006.pdf]

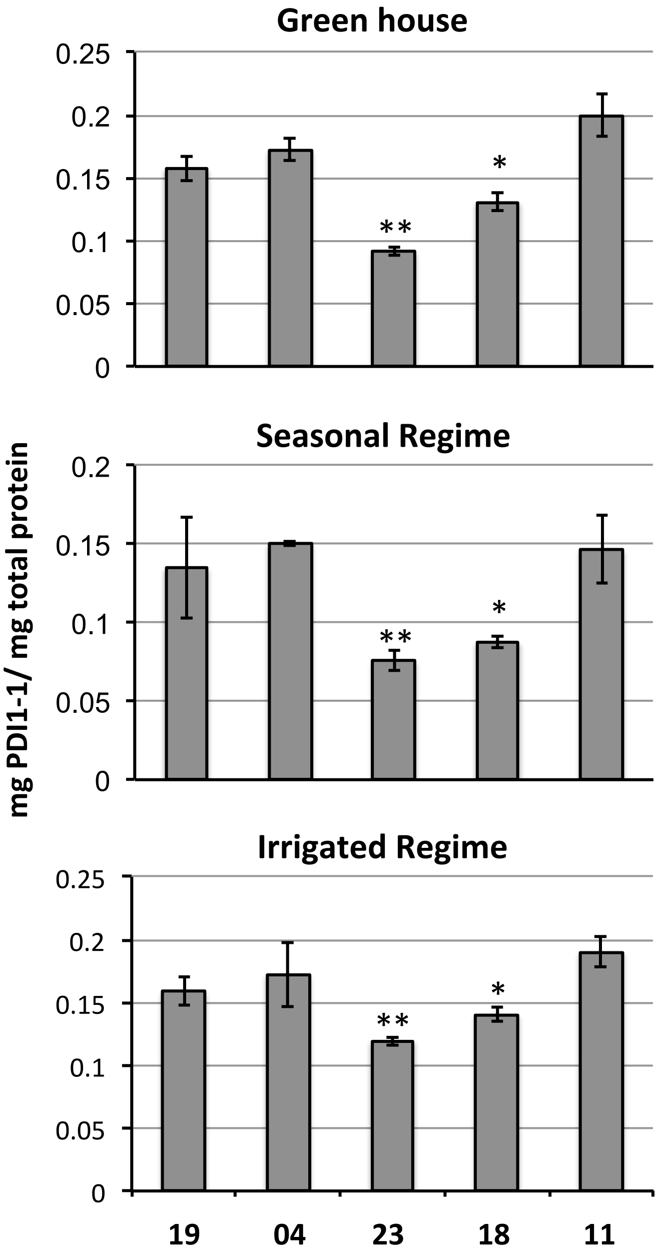

S6 Fig. PDI1-1 levels in the mature seeds of five Mexican barley cultivars (19, 04, 23, 18 and 11) under different growth conditions. Proteins were extracted from whole seeds, PDI1-1 quantified by ELISA and normalized to total protein in three independent protein extractions for each growth condition (green house; seasonal regime in the field; irrigated regime in the field). Significant differences were found for cultivars 23 (**; $p<0.01$) and 18 (*; $p < 0.05$).
